# The prognostic role of EGFR-TKIs for patients with advanced non-small cell lung cancer

**DOI:** 10.1038/srep40374

**Published:** 2017-01-12

**Authors:** Dan Zhao, Xuejing Chen, Na Qin, Dan Su, Lijuan Zhou, Quan Zhang, Xi Li, Xinyong Zhang, Mulan Jin, Jinghui Wang

**Affiliations:** 1 Department of Pathology, Beijing Chest Hospital, Capital Medical University, Beijing Tuberculosis and Thoracic Tumor Research Institute, China; 2 Department of Medical Oncology, Beijing Chest Hospital, Capital Medical University, Beijing Tuberculosis and Thoracic Tumor Research Institute, Beijing, China; 3 Department of Pathology, Beijing Chao-yang Hospital, Capital Medical University, Beijing, China

## Abstract

Clinical trials have shown that epidermal growth factor receptor-tyrosine kinase inhibitors (EGFR-TKIs) did not improve the survival of patients with EGFR-mutated non-small cell lung cancer (NSCLC) because of the high crossover of treatments. Realistically, the role of EGFR-TKIs in NSCLC with mutated EGFR is not well known. We retrospectively analysed data from patients with recurrent or metastatic NSCLC. Clinical prognostic factors were identified by Cox proportional hazards modelling. Among 503 patients, the median overall survival (OS) for all of patients was 11.7 months. Cox analysis showed that PS 0–1, recurrent disease, EGFR mutations, or EGFR-TKI treatment were associated with improved OS. In patients with EGFR-activating mutations, Cox analysis showed that patients with adenocarcinoma, recurrent disease, or EGFR-TKI treatment had significantly longer survival. Patients with EGFR-activating mutations who received EGFR-TKI therapy had a median OS of 24.3 months, which was significantly longer than those who had not received EGFR-TKI therapy (10.8 months). Patients with wild-type EGFR had a median OS of 9.7 months and Cox analysis showed that PS score and disease type were independent predictors. EGFR-TKI therapy is an independently prognostic factor for NSCLC with mutated EGFR. A more effective therapy is needed for patients with wild-type EGFR.

Lung cancer is the leading cause of cancer death worldwide. Approximately 75% of patients diagnosed with advanced disease have a dismal prognosis. Chemotherapy has been the most important modality for advanced or recurrent non-small cell lung cancer (NSCLC) but only achieves a median survival of 8–10 months. The immense progress in treatment options, including the development of epidermal growth factor receptor (EGFR)-tyrosine kinase inhibitors (TKIs), has changed the modality of treatment for NSCLC harbouring EGFR-activating mutations. The first-generation TKIs gefitinib and erlotinib, the second-generation TKI afatinib, and the third-generation TKI osmertinib have been approved by the U.S. Food and Drug Administration (FDA) for use in clinical settings. Icotinib, a type of first-generation TKI, has been approved by the China Food and Drug Administration (CFDA). The frequency of EGFR mutations in lung cancer in Caucasian is 17%^1^; in American lung adenocarcinoma populations, the frequency is 23%^2^; and in Chinese lung adenocarcinoma patients, it is 51%^3^. Patients are routinely tested for these mutations in clinical practice.

A series of randomized clinical trials on EGFR-TKIs for patients with EGFR-activating mutations have demonstrated that EGFR-TKIs are the most effective therapy, with distinct prolonged, progression-free survival of approximately 9.2–13.7 months^4^,^5^,^6^,^7^,^8^,^9^,^10^,^11^. Patients had a median overall survival (OS) of 19.3 to 35.5 months. These trials have not demonstrated that EGFR-TKIs can improve the OS for patients with EGFR-mutated NSCLC compared with chemotherapy because of the crossover treatment of the two groups. A meta-analysis published by Lee *et al*.^12^ also showed that EGFR-TKI therapy significantly delays disease progression in patients with EGFR mutations but has no demonstrable impact on OS; treatment with EGFR-TKIs had no impact on OS for patients with mutated-EGFR or wild-type EGFR. Compared with platinum-based chemotherapy, afatinib, a second-generation TKI, did not improve OS in an entire population with EGFR-sensitive mutations but improved OS for patients with del19 EGFR mutations^13^. The prognostic role of EGFR-TKIs in patients with EGFR-mutations is not known. Thus, we retrospectively analysed data of patients with an identified EGFR status and explored the prognostic factors of survival, including EGFR-TKI therapy, for patients with NSCLC.

## Results

### Patient characteristics

In total, 503 patients with NSCLC were enrolled in this study. The median age was 59 years old (range, 21–86 years old). There were 293 male patients (58.3%) and 210 female patients (41.7%). There were 243 non-smokers (48.3%), 259 smokers (51.5%), and 1 patient (0.2%) for which there was no record on smoking history. There were 435 (86.5%) patients with adenocarcinoma, 58 (11.5%) with squamous cell carcinoma, 4 (0.8%) with NSCLC not otherwise specified (NOS), 2 (0.4%) with large cell carcinoma, and 4 (0.8%) with a mixed type. There were 135 (26.8%) patients with recurrent disease and 368 (73.2%) patients with locally advanced and metastatic disease (59 locally advanced and 309 metastatic) (Table 1).

### EGFR genotype

Among all 503 patients, the incidence of EGFR mutations was 36.6%; 184 patients had EGFR mutations, and 319 patients were wild type. Of the 184 patients with EGFR mutations, 86 patients (46.7%) had exon 19 deletions (del19), 81 patients (44.0%) had an L858R mutation at exon 21, 4 patients (2.2%) had exon 18 mutations, 5 patients (2.7%) had an L861Q mutation at exon 21, 1 patient (0.5%) had an exon 20 insertion, 2 patients (1.1%) had a T790M mutation at exon 20, and 5 patients (2.7%) had multiple mutations, of which there were 2 patients with del19 and L858R mutations, 2 with T790M and L858R mutations, and 1 with an L861Q and an L858R mutation. Based on histological type, 40.7% (177/435) of lung adenocarcinoma patients and 10.3% (6/58) of lung squamous cell carcinoma patients had EGFR mutations.

For statistical purposes, EGFR status was defined as activating mutations or wild type. Patients with activating mutations included 86 with del19, 81 with L858R at exon 21, 4 with G719X at exon 18, 5 with L861Q at exon 21, and 3 with multiple activating mutations. However, 1 patient with an exon 20 insertion, 2 patients with a T790M mutation, and 2 patients with combined activating and resistant mutations were excluded. Overall, 179 patients had activating EGFR mutations and 319 patients were wild type.

### Treatment

Overall, 392 patients (77.9%) received systemic treatment, including 228 patients (58.2%) who received platinum-based combined chemotherapy, 36 patients (9.2%) who received single-agent chemotherapy, 108 patients (27.6%) who received EGFR-TKIs, and 20 patients (5.1%) who received chemotherapy plus anti-vascular drugs as a first-line treatment. In total, 244 patients received EGFR-TKI therapy, including 108 (44.3%) who received it as a first-line treatment, 100 (41.0%) as a second-line treatment, 24 (9.8%) as a third-line treatment, 9 (3.7%) as a fourth-line treatment, and 3 (1.2%) were others.

Of the 179 patients with EGFR-activating mutations, 146 patients (81.6%) received systemic treatment, including 65 patients (44.5%) who received EGFR-TKIs, 66 patients (45.2%) who received combined chemotherapy, 11 (7.5%) who received single-agent chemotherapy, and 4 patients (2.7%) who received chemotherapy plus anti-vascular drugs as a first-line treatment. In addition, there were 33 patients (18.4%) who did not received systemic anti-tumour treatments. Of the patients with EGFR-activating mutations, 117 patients received EGFR-TKI treatment, including 65 (55.6%) who received it as a first-line treatment, 42 (35.9%) as a second-line treatment, 7 (6.0%) as a third-line treatment, 1 (0.9%) as a fourth-line treatment, and 2 (1.7%) were others.

Of the 319 patients with wild-type EGFR, 242 patients (75.9%) received first-line treatment, including 158 patients (65.3%) who received combined chemotherapy, 25 (10.3%) who received single-agent chemotherapy, 43 (17.8%) who received EGFR-TKIs, and 16 (6.6%) who received chemotherapy plus anti-vascular drugs as a first-line treatment. In addition, 77 patients (24.1%) did not receive systemic anti-tumour treatment.

### Survival of all of patients

All of the patients were followed until December 31, 2015. In total, 421 patients (83.7%) died (133 of 184 with EGFR mutations and 288 of 319 with wild-type EGFR), and 82 patients (16.3%) were alive at the end of the study (51 of 184 with EGFR mutations and 31 of 319 with wild-type EGFR). The median OS was 11.7 months (95% CI 10.521 to 12.879).

**Figure 1:**
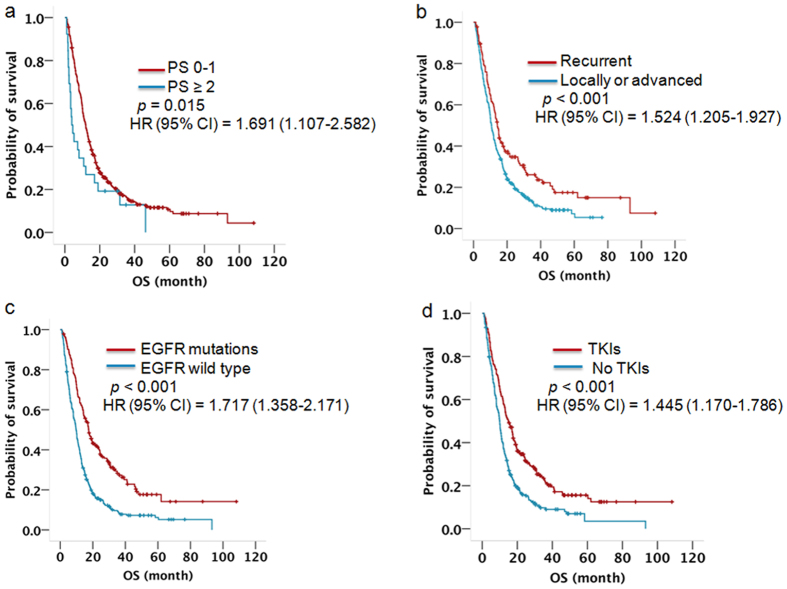
Kaplan-Meier survival curve for the 503 patients stratified by (**a**) PS score, (**b**) disease type, (**c**) EGFR status, (**d**) EGFR TKI therapy. P-value indicates significance levels from the comparison of survival curves using the Log-rank test.

In a univariate analysis, clinicopathological characteristics, specifically, age, gender, smoking status, PS score, histological type, stage, and EGFR status, as well as whether they received EGFR-TKI therapy, were analysed. The results showed that patients with specific characteristics (female, non-smoking, PS of 0–1, adenocarcinoma, recurrent disease, EGFR mutations, receiving EGFR-TKI therapy) had a significantly longer survival than patients with opposing characteristics (male, smoker, PS ≥ 2, squamous cell carcinoma, locally advanced and metastatic disease, wild-type EGFR, not receiving EGFR-TKI therapy) (Table 2). These significant variables were enrolled for a Cox analysis. A good PS score (Hazard Ratio 1.691, 95% CI 1.107 to 2.582, *p* = 0.015, Fig. 1a), recurrent disease (Hazard Ratio 1.524, 95% CI 1.205 to 1.927, *p* < 0.001, Fig. 1b), EGFR mutations (Hazard Ratio 1.717, 95% CI 1.358 to 2.171, *p* < 0.001, Fig. 1c), and receiving EGFR-TKI therapy (Hazard Ratio 1.445, 95% CI 1.170 to 1.786, *p* < 0.001, Fig. 1d) were independent predictors of OS for all patients with NSCLC (Table 2).

### Survival for patients with EGFR-activating mutations

**Figure 2:**
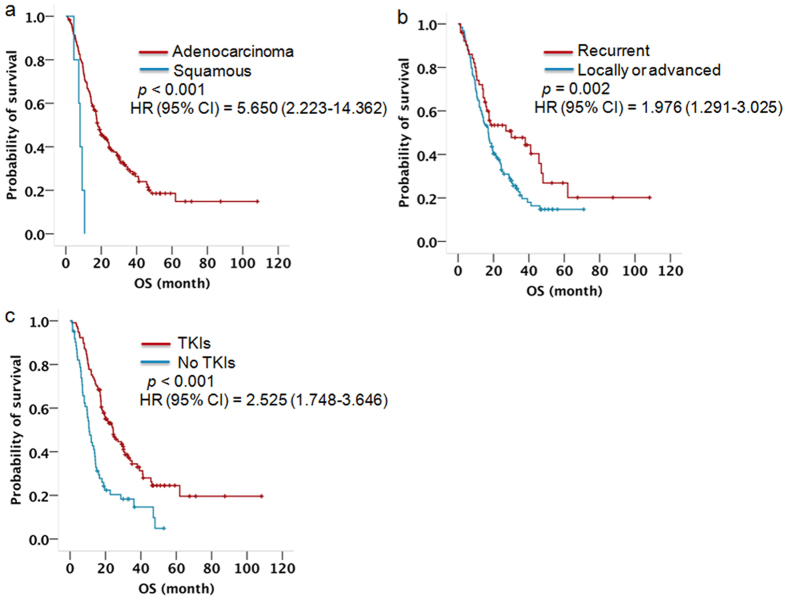
Kaplan-Meier survival curve for patients with activating EGFR mutations stratified by (**a**) histological type, (**b**) disease type, (**c**) EGFR TKI therapy. P-value indicates significance levels from the comparison of survival curves using the Log-rank test.

Of the 179 patients with EGFR-activating mutations, 129 (72.1%) died and 50 (27.9%) were alive at the end of the study. The median survival for these patients was 17.5 months (95% CI 15.055 to 19.945). Of these patients, 117 received EGFR-TKI therapy as different treatment lines. Gender, histological type, disease type, or treatment with EGFR-TKIs were enrolled in a univariate analysis, and the results showed that the median OS of patients who were female, or had adenocarcinoma or recurrent disease, or received EGFR-TKI therapy had a significantly longer OS than patients who were male, or had squamous or metastatic disease, or were not treated with EGFR-TKIs (Table 3). There were no differences in the survival of patients related to age, smoking status, or PS score. In a multivariate analysis, patients with adenocarcinoma (Hazard Ratio 5.650, 95% CI 2.223 to 14.362, *p* < 0.001, Fig. 2a), recurrent disease (Hazard Ratio 1.976, 95% CI 1.291 to 3.025, *p* = 0.002, Fig. 2b), or receiving EGFR-TKI therapy (Hazard Ratio 2.525, 95% CI 1.748 to 3.646, *p* < 0.001, Fig. 2c) had a significantly longer survival than those with squamous cell carcinoma, metastatic disease, or without EGFR-TKI therapy (Table 3).

For the 117 patients harbouring EGFR-activating mutations and who received EGFR-TKIs, their median OS was 24.3 months (95% CI 18.076 to 30.524). There was no survival difference found in relation to age, gender, smoking status, PS score, disease type, mutation type, or line of EGFR-TKI therapy. Cox analysis showed that only histological type was an independent factor of OS, and adenocarcinoma patients had a better survival rate than squamous cell carcinoma patients (Hazard Ratio 11.984, 95% CI 3.873 to 37.082, *p* < 0.001) (Table 4). # 7 patients with third-line TKIs, 1 patient with fourth-line TKIs, and 2 with other lines were not enrolled.

### Survival for patients with wild-type EGFR

**Figure 3:**
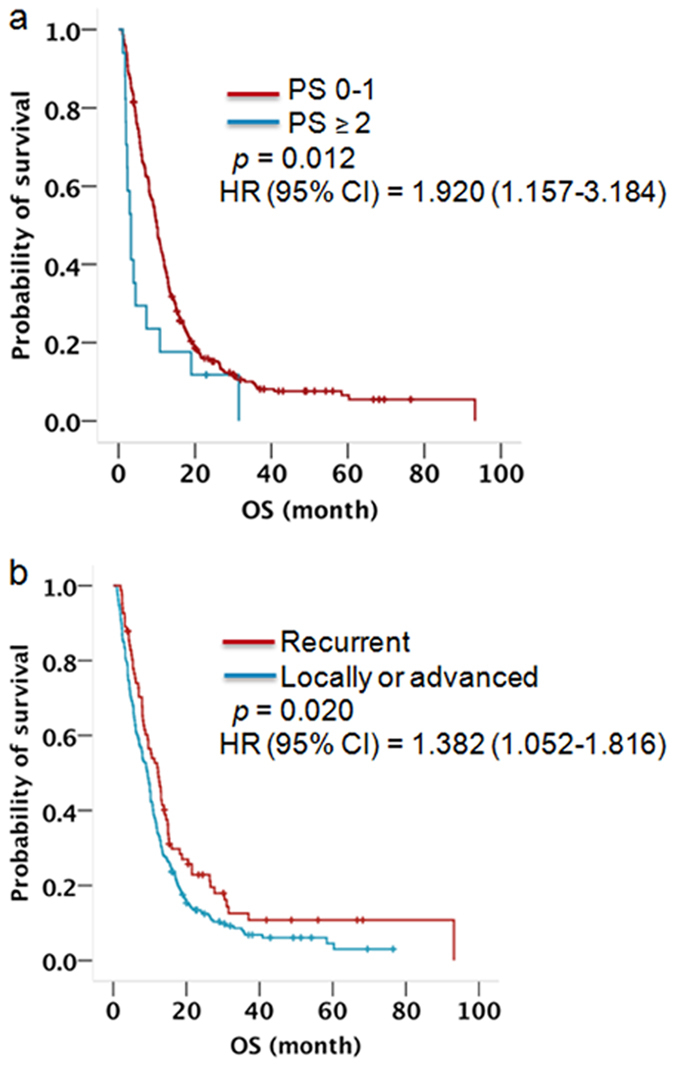
Kaplan-Meier survival curve for patients with wild-type EGFR stratified by (**a**) PS score, (**b**) disease type. P-value indicates significance levels from the comparison of survival curves using the Log-rank test.

Of the 319 patients with wild-type EGFR, 288 (90.3%) had died. The median OS for these patients was 9.7 months (95% CI 8.506 to 10.894). Univariate analysis showed that patients with PS 0–1 and recurrent disease had a significantly longer survival. There were no survival differences related to age, gender, smoking status, histological type, or EGFR-TKI therapy. Multivariate analysis showed that a PS of 0–1 (Hazard Ratio 1.920, 95% CI 1.157 to 3.184, *p* = 0.012, Fig. 3a) and recurrent disease (Hazard Ratio 1.382, 95% CI 1.052 to 1.816, *p* = 0.020, Fig. 3b) were independent predictors of OS (Table 5).

## Discussion

We collected clinical data from the Beijing Chest Hospital and aimed to analyse prognostic factors for advanced NSCLC in patients with different EGFR status and identify the role of EGFR-TKIs in improving OS for patients with EGFR mutations. The median OS of all of patients was 11.7 months (95% CI 10.521 to 12.879). For patients with EGFR-activating mutations, receiving EGFR-TKI therapy resulted in a significantly longer survival than those without EGFR-TKI therapy (Hazard Ratio 2.525, 95% CI 1.748 to 3.646, *p* < 0.001).

As far as we know, typically, certain characteristics, such as a PS of 0–1, female, and adenocarcinoma are good indicators of longer survival for all patients with NSCLC. In our study, a univariate analysis showed that specific characteristics (female, non-smoking, PS 0–1, adenocarcinoma, recurrent disease, EGFR mutations, receiving EGFR-TKI therapy) predicted a better survival outcome. However, the multivariate analysis showed that a good PS score (Hazard Ratio 1.691, 95% CI 1.107 to 2.582, *p* = 0.015), recurrent disease (Hazard Ratio 1.524, 95% CI 1.205 to 1.927, *p* < 0.001), EGFR mutations (Hazard Ratio 1.717, 95% CI 1.358 to 2.171, *p* < 0.001), or receiving EGFR-TKI therapy (Hazard Ratio 1.445, 95% CI 1.170 to 1.786, *p* < 0.001) were independent factors of OS for all patients with NSCLC. Because several studies have demonstrated that female patients and adenocarcinoma patients have a higher frequency of EGFR mutations, EGFR status may be a valuable factor to predict survival, rather than gender or histological type.

For all of patients harbouring EGFR-activating mutations, the median OS was 17.5 months (95% CI 15.055 to 19.945), and a Cox analysis showed that adenocarcinoma (Hazard Ratio 5.650, 95% CI 2.223 to 14.362, *p* < 0.001), recurrent disease (Hazard Ratio 1.976, 95% CI 1.291 to 3.025, *p* = 0.002), or treatment with EGFR-TKIs (Hazard Ratio 2.525, 95% CI 1.748 to 3.646, *p* < 0.001) were associated with improved OS. For patients harbouring EGFR-activating mutations who received EGFR-TKI therapy, the OS was significantly higher than that of patients harbouring EGFR-activating mutations who did not receive EGFR-TKIs (median survival, 24.3 vs. 10.8 months, respectively; *p* < 0.001). Our result regarding the survival of patients with EGFR-mutations who received EGFR-TKIs was different form previous studies^4^,^5^,^6^,^7^,^8^,^9^,^10^,^11^. The present study showed that EGFR-TKIs can prolong the OS of patients with EGFR-mutations compared with those did not receive TKIs, indicating that patients with EGFR-activating mutations should receive EGFR-TKI therapy.

To analyse the prognostic factors of survival for patients harbouring activating mutations who received EGFR-TKIs, multiple factors including age, gender, smoking status, PS score, histological type, disease type, activating mutation type, and lines of treatments were enrolled for univariate and multivariate analysis, and the results showed that there was a significant difference in survival between patients with lung adenocarcinoma and patients with lung squamous cell carcinoma (24.5 months vs. 7.3 months, Hazard Ratio 11.984, 95% CI 3.873 to 37.082, *p* < 0.001). The prognostic role of EGFR mutations in squamous cell carcinoma had rarely been reported. Han *et al*. reported that, among 29 EGFR-positive patients with squamous lung cancer (which was a small sample size for the retrospective analysis), EGFR mutation-positive patients had significantly improved OS with EGFR-TKI therapy compared with those who did not receive EGFR-TKIs (18.04 months [95% CI 13.47 to 22.61] vs. 13.18 months [95% CI 5.22 to 21.13], *p* = 0.015)^14^. Two meta-analyses showed that survival for patients with del19 was superior to patients with an L858R mutation, all of whom received EGFR-TKI therapy^15^,^16^. Our data were not consistent with previous studies. There are several possible reasons. First, most importantly, the censored number was slightly higher in the del19 group, among 179 patients, there were 86 patients harbouring del19 and 81 patients harbouring an L858R mutation. Before the end of the study, 59 (68.6%) of 86 patients with del19 and 63 (77.8%) of 81 patients with L858R died. Second, the overall treatment of the two groups was not very consistent. Third, patients had a median survival of 24.5 months (95% CI 20.524 to 28.476) for the del19 group compared with 21.6 months (95% CI 7.620 to 35.580) for the L858R group, and these data showed a trend.

We also performed a multivariate analysis of OS for patients with wild-type EGFR NSCLC in our large sample size study. Patients with wild-type EGFR had a poor median OS of 9.7 months (95% CI 8.506 to 10.894). Patients with a good PS or recurrent disease survived longer. Whether in non-selected patients, or in patients with EGFR mutations or wild-type EGFR patients, OS for patients with recurrent disease was superior to patients with locally advanced or metastatic disease. The most important reason is that tumour load in patients with recurrent disease is much lower than that of advanced patients, and this directly influences OS. For patients with wild-type EGFR, who had a rare frequency of oncogene, chemotherapy alone or chemotherapy combined with anti-vascular drugs is the standard regimen. However, the development of new and more effective treatment is urgent.

Because this was a retrospective analysis, some tissues were specifically checked for EGFR mutations; some EGFR wild-type patients received EGFR-TKIs without detection of EGFR. In 319 patients with wild-type EGFR, 126 patients (29.5%) had received EGFR-TKI therapy. The median OS of those who received EGFR-TKI therapy (10.2 months, 95% CI 8.079 to 12.321) was similar to that of patients who had not received EGFR-TKIs (9.5 months, 95% CI 8.150 to 10.850) (*p* = 0.320). Our results also indicated that wild-type patients would not benefit from EGFR-TKI therapy, which is similar to the result of the previous study^4^.

Kris *et al*.^17^ reported that patients with EGFR mutations in America had a median survival of 3.97 years. In his study, the survival of patients with cancer-driving genes who received targeted therapy was superior to those who had driving genes but were not treated with targeted therapy, as well as to those without driving genes. The data in our study indicated a shorter survival time than Kris’s results, and we think there were several reasons for the difference. First, EGFR-TKIs are not covered by medical insurance in most provinces in China, which limits the use of targeted therapy and lowers patient compliance. In recent years, EGFR testing is standard for advanced treatment-naïve patients in most cancer centres or big comprehensive hospitals in China, and more and more patients with EGFR mutations have been treated with first-line EGFR-TKI therapy. Second, many of the new targeted drugs, for example, second and third-generation EGFR-TKIs, are not available in China, which influences the subsequent treatment of patients who did not respond to first-generation EGFR-TKIs. Third, patients in China tend to have a more serious, advanced stage of the disease, which influences the efficacy of EGFR-TKI treatment and survival.

There are several limitations in this study. First, this is a retrospective study, and thus, there is a bias of patients to some degree. Second, the methods used to detect EGFR mutations and the treatment process were not very uniform.

In conclusion, our study showed that treatment with EGFR-TKIs is an independent predictor for patients with EGFR-mutated NSCLC. NSCLC patients with EGFR-activating mutations who received EGFR-TKI therapy had significantly longer survival than those without EGFR-TKI therapy. The survival of patients with wild-type EGFR was slightly shortened. Therefore, more molecular-basis research is needed to further develop more effective regimens.

## Methods

### Study design and patients

We aimed to conduct a retrospective analysis of OS for patients with an identified EGFR mutation status in a single cancer hospital, and we analysed the prognostic role of EGFR-TKIs in the OS of patients with different EGFR statuses. All patients with NSCLC who had an identified EGFR status and who were treated at Beijing Chest Hospital from January 2006 to December 2014 were enrolled retrospectively in this study. The inclusion criteria were as follows: all of the patients in the study were diagnosed with NSCLC by histology. EGFR status was tested using tumour tissues or cellblocks of confirmed malignant pleural effusion. All of the patients had a follow-up to assess OS. The exclusion criteria were as follows: patients without EGFR status testing or loss of follow-up were excluded. Written informed consent was obtained from each patient. The study was approved by the institutional review board of Beijing Chest Hospital and conducted according to the guidelines approved by the ethics committee.

### Data collection

Medical records of all patients were reviewed and clinicopathological factors, including age, gender, performance status (PS) score, smoking history, histological type, disease type, EGFR mutation type, and treatment, as well as EGFR-TKI therapy, were recorded. Trained professional staff in our hospital collected follow-up data. PS score was determined according to the definition of the Eastern Cooperative Oncology Group (ECOG). Non-smoking was defined as patients who had smoked less than 100 cigarettes in their life. Histological type was identified by WHO criteria^18^. TNM staging was performed according to the 7th edition of the American Joint Committee for Cancer (AJCC) staging system^19^. EGFR detection methods included DNA direct sequencing and an amplification refractory mutation system (ARMS). The methods were performed in accordance with the approved guidelines.

### Statistical analysis

Kaplan-Meier method was used to analyse OS, and a log-rank test was used to compare difference between two groups. OS for locally advanced or metastatic disease was calculated from the date of diagnosis to the date of death due to any cause, and OS for recurrent patients was calculated from the date of recurrence after surgery to the date of death due to any cause. Patients who had not died as of the data cutoff date were censored. A Cox proportional hazards regression model was used to identify independent factors of OS. A two-sided *p*-value < 0.05 was considered statistically significant. All statistical analyses were conducted on SPSS 22.0 software.

## Additional Information

**How to cite this article:** Zhao, D. *et al*. The prognostic role of EGFR-TKIs for patients with advanced non-small cell lung cancer. *Sci. Rep.* **7**, 40374; doi: 10.1038/srep40374 (2017).

**Publisher's note:** Springer Nature remains neutral with regard to jurisdictional claims in published maps and institutional affiliations.

## Figures and Tables

**Table 1:** Basic characteristic of 503 patients.

Characteristics	N	%
Age		
Median	59	
Range	21–86	
Gender		
Male	293	58.3
Female	210	41.7
Smoking status		
Non-smokers	243	48.3
Smoking	259	51.5
No record	1	0.2
PS score		
0–1	477	94.8
≥2	26	5.2
Histological type		
Adenocarcinoma	435	86.5
Squamous	58	11.5
NSCLC NOS	4	0.8
Large cell lung cancer	2	0.4
Mixed type	4	0.8
Disease type		
Recurrent	135	26.8
Locally advanced or metastatic disease	368	73.2
EGFR		
Mutation	184	36.6
Wild type	319	63.4

**Table 2:** Univariate and multivariate analysis of survival for 503 patients.

	N	Events	Univariate			Multivariate	
			OS (month)	95% CI	*p*	*p*	HR (95% CI)
Age							
≤65	353	292	11.7	10.385–13.015	0.554		
>65	150	129	11.0	8.750–13.250			
Gender							
Male	293	256	10.3	9.047–11.553	0.004	0.934	1.011 (0.773–1.323)
Female	210	165	12.7	10.581–14.819			
Smoking status							
Non-smoking	259	205	12.4	10.420–14.380	0.001	0.298	0.868 (0.664–1.133)
Smoking	243	216	10.6	9.193–12.007			
PS score							
0–1	477	398	12.0	10.846–13.154	0.024	0.015	1.691 (1.107–2.582)
≥2	26	23	4.0	1.626–6.374			
Histological type*							
Adenocarcinoma	435	359	12.0	10.656–13.344	0.033	0.784	1.045 (0.762–1.434)
Squamous	58	32	8.3	6.336–10.264			
Disease type							
Recurrent disease	135	101	14.2	12.450–15.950	0.001	<0.001	1.524 (1.205–1.927)
Local or metastatic disease	368	320	10.7	9.782–11.618			
EGFR							
Mutation	184	133	17.5	15.055–19.945	<0.001	<0.001	1.717 (1.358–2.171)
Wild type	319	288	9.7	8.506–10.894			
EGFR TKI therapy							
Yes	244	192	14.8	12.3346–17.254	<0.001	<0.001	1.445 (1.170–1.786)
No	259	229	10.0	8.958–11.042			

P values were listed in the table. *4 patients with NSCLC NOS, 2 patients with large cell lung cancer NOS, and 4 patients with mixed type were not enrolled.

**Table 3:** Univariate and multivariate analysis of survival for 179 patients with an EGFR-activating mutation.

	N	Events	Univariate			Multivariate	
			OS (month)	95% CI	*p*	*p*	HR (95% CI)
Age							
≤65	140	101	17.5	14.978–20.022	0.927		
>65	39	28	14.3	7.446–21.154			
Gender							
Male	78	60	16.1	12.891–19.309	0.059		
Female	101	69	19.6	14.396–24.804			
Smoking status							
Non-smoking	125	90	17.5	14.222–20.778	0.502		
Smoking	54	39	19.0	15.203–22.797			
PS score							
0–1	170	122	17.8	15.363–20–20.237	0.469		
≥2	9	7	12.0	0.605–23.395			
Histological type*							
Adenocarcinoma	173	123	18.0	15.097–20.903	<0.001	<0.001	5.650 (2.223–14.362)
Squamous	5	5	7.3	6.497–9.503			
Disease type							
Recurrent disease	51	31	30.1	3.685–56.515	0.028	0.002	1.976 (1.291–3.025)
Local or metastatic disease	128	98	17.0	13.564–20.436			
EGFR TKI therapy							
Yes	117	77	24.3	18.076–30.524	<0.001	<0.001	2.525 (1.748–3.646)
No	62	52	10.8	8.397–13.203			

P values were listed in the l table. *1 patient with large cell lung cancer was not enrolled.

**Table 4:** Univariate and multivariate analysis of 117 patients with an EGFR-activating mutation who received EGFR TKI therapy.

	N	Events	Univariate			Multivariate	
			OS (month)	95% CI	*p*	*p*	HR (95% CI)
Age							
≤65	93	62	24.4	19.743–29.057	0.992		
>65	24	15	19.6	3.851–35.349			
Gender							
Male	46	32	23.6	15.857–31.343	0.290		
Female	71	45	24.5	14.740–34.260			
Smoking status							
Non-smoking	84	56	24.3	17.741–30.859	0.844		
Smoking	33	21	25.8	16.049–35.551			
PS score							
0–1	109	71	24.4	17.741–31.059	0.294		
≥ 2	8	6	8.1	0.0–24.454			
Histological type							
Adenocarcinoma	113	73	24.5	17.538–31.462	<0.001	<0.001	11.984 (3.873–37.082)
Squamous	4	4	7.3	3.870–10.730			
Disease type							
Recurrent disease	30	17	38.0	20.526–55.474	0.093		
Local or metastatic disease	87	60	21.6	16.819–26.381			
Mutation type*							
19 Del	58	38	24.5	20.524–28.476	0.519		
L858R	47	32	21.6	7.620–35.580			
Line of TKI therapy#							
First line	65	37	19.6	13.213–25.987	0.903		
Second line	42	33	24.4	19.243–29.557			

P values were listed in the Table. *4 patients with exon 18 mutations, 5 patients with a L861Q mutation, and 3 patients with other types were not enrolled. ^#^7 patients with third-line TKIs, 1 patient with fourth-line TKIs, and 2 with other lines were not enrolled.

**Table 5:** Univariate and multivariate analysis of survival for 319 patients with wild-type EGFR.

	N	Events	Univariate			Multivariate	
			OS (month)	95% CI	*p*	*p*	HR (95% CI)
Age							
≤65	210	189	9.7	8.349–11.051	0.541		
>65	109	99	9.8	8.219–11.381			
Gender							
Male	212	194	9.7	8.588–10.812	0.861		
Female	107	94	9.8	7.284–12.316			
Smoking status							
Non-smoking	132	113	9.7	8.357–11.043	0.143		
Smoking	187	175	9.9	8.432–11.368			
PS score							
0–1	302	272	10.0	9.024–10.976	0.009	0.012	1.920 (1.157–3.184)
≥ 2	17	16	3.3	2.107–4.493			
Histological type*							
Adenocarcinoma	258	233	9.7	8.654–10.746	0.807		
Squamous	52	46	9.9	7.233–12.567			
Disease type							
Recurrent disease	82	69	12.3	9.855–14.745	0.018	0.020	1.382 (1.052–1.816)
Local or metastatic disease	237	219	9.4	8.232–10.568			
EGFR TKI therapy							
Yes	126	114	10.2	8.079–12.321	0.320		
No	193	174	9.5	8.150–10.850			

P values were listed in the table. *4 patients with NSCLC NOS, 1 patient with large cell lung cancer, and 4 patients with mixed type were not enrolled.
